# From learning to reversal learning: How non-cleaner fish tackle the biological market task

**DOI:** 10.1007/s10071-025-01983-w

**Published:** 2025-07-24

**Authors:** Laurent Prétôt, Hannah Miller, Kayla Leyden

**Affiliations:** 1 Department of Psychology and Counseling, Pittsburg State University, Pittsburg, KS 66762 U.S.A.; 2 SEA LIFE Aquarium Kansas City, Kansas City, MO 64108 U.S.A.

**Keywords:** Ecological approach, Decision-making, Reversal learning, Biological market task, Ephemeral reward task, Dottyback, *Pseudochromis*

## Abstract

The biological market task – also known as the ephemeral reward task – models the mutualistic cleaning interactions between bluestreak cleaner wrasses and their client fish on coral reefs. In this dichotomous choice paradigm, selecting an “ephemeral” food option first grants access to a “permanent” food option, while choosing the permanent option first makes the ephemeral one unavailable. Cleaner fish have previously outperformed other vertebrates on this task, presumably because the cues to solve it are more ecologically salient for cleaner fish. In this study, we tested whether this advantage extends to non-cleaner fish by assessing the learning and reversal learning performance of three dottyback species (*Pseudochromis* spp.) – mesopredator reef fish that do not engage in cleaning mutualisms – on the original task and two derived versions that varied in the cue required for solving it. Dottybacks performed poorly in all versions of the task. Notably, they did worse in the original task than cleaner wrasses tested previously, suggesting that cleaner fish’ success is tied to specific ecological conditions not shared by other species. Further analyses revealed subtle differences in performance between tasks and faster learning in the initial test compared to the reversal test, an indicator of limited cognitive flexibility. Together, these findings help fill a gap in the biological market literature and underscore how species-specific ecological traits and task structure shape cognitive performance.

**Supplementary Information:**

The online version contains supplementary material available at 10.1007/s10071-025-01983-w.

## Introduction

The ecological approach to cognition explains within- and between-species variation in cognitive performance as a result of differences in ecological adaptation (Kamil 1998; Shettleworth 2010). In contrast to the traditional view on cognition – which posits a relationship between brain size and cognitive performance yet often fails to establish a consistent correlation (see Chittka and Niven 2009; Healy and Rowe 2007) – the ecological approach predicts success based on species’ differences in sensitivity to specific cues, which they rely on to solve ecological problems. Among its major contribution to the field of animal cognition, the ecological approach has allowed the integration of studies on a wider range of animals and the appreciation that even species with small and less sophisticated brains can outperform others in tasks for which they have evolved specialized adaptations. Among taxa that have benefited the most from the ecological approach, fish remain relatively underused (Bshary and Triki 2022). Yet, fish have abundant continuous neurogenesis (Schmidt et al. 2013) and neuronal regeneration capacities (Zupanc 2006), along with highly plastic brains that allow them to adjust cognitively to environmental changes (Kotrschal and Taborsky 2010; Lucon-Xiccato et al. 2023; Montalbano et al. 2022; Salvanes et al. 2013), thus making them a great fit for testing the ecological approach.

A well-known example of ecological adaptation in fish is found in the mutualistic cleaning interaction between bluestreak cleaner wrasses *Labroides dimidiatus* and their various client fish species, who live on coral reefs in the tropics from the Red Sea and Indian Ocean to the western Pacific Ocean. In this interaction, the client fish visits the cleaner fish’s territory to have its ectoparasites and infected or dead tissue removed. Importantly, clients have been categorized as either ephemeral (also called visitor or choosy), which home range covers several cleaning stations and thus gives them access to several cleaner fish, or permanent (also called resident), who live on a single coral reef patch and have access to a single cleaner fish. Observations in the wild indicate that ephemeral clients receive priority for cleaning access over permanent clients (Bshary 2001), presumably because they are more likely to switch to another cleaner fish if ignored and more likely to return if they are inspected (Bshary and Schäffer 2002). Bshary and Grutter (2002) simulated this exact dilemma in the laboratory – known as the *biological market task * – by replacing the clients with two different plates, each carrying one food item. In the experiment, eating the food from the *ephemeral* plate first gives the subject access to the food from the *permanent* plate (for a total of two rewards), whereas eating the food from the permanent plate first leads to the loss of the ephemeral food (for a total of one reward). Therefore, choosing the ephemeral stimulus first represents the food-maximizing (optimal) choice, whereas choosing the permanent stimulus first represents the suboptimal choice. In the task, cleaner fish quickly learned to prioritize the ephemeral plate over the permanent plate, thus aligning with previous observations in nature.

Despite the apparent simplicity of the original plate task, choosing optimally has proven to be challenging for a wide range of animals tested so far, including non-human primates (e.g., tufted capuchins, drills, gorillas, orangutans, chimpanzees; Prétôt et al. 2020; Salwiczek et al. 2012), pigeons (Zentall et al. 2016), rats (Zentall et al. 2017b), and even humans (Mueller et al. 2024). However, the task can be solved with procedural modifications, including changing the cues to be more salient (Prétôt et al. 2016a, b), controlling for extraneous cues (Prétôt et al. 2016a), delaying the reward following the choice (Zentall et al. 2017a, b), and reducing the probability of reward for choosing either option (Peng et al. 2025). Interestingly, grey parrots (*Psittacus erithacus*) remain the only species tested so far that performed at levels similar to cleaner fish in the original experiment (Pepperberg and Hartsfield 2014). The authors proposed that the task might be particularly natural for parrots and fish, because both are naturally accustomed to sequential acquisition of food by making only one single choice at a time with their bill or mouth.

Intriguingly, only a handful of studies to date have examined the performance of other fish species in the task. Salwiczek and colleagues (2012) first reported that *Labroides bicolor * – a cleaner wrasse species closely related to *L. dimidiatus * – performed more poorly than its congener in the original plate task, presumably because of differences in the way both species approach their clients. More recently, Ranucci et al. (2024) found that cleaner gobies (*Elacatinus oceanops*) could solve the task when the plates differed in size, a cue associated with increased parasite loads (Soares et al. 2007). An important remaining question, however, concerns the extent to which these results *generalize* to non-cleaner fish. Here, we begin to investigate this question by testing three non-cleaner fish species from the genus *Pseudochromis* – *P. aldabraensis*, *P. flavivertex*, and *P. fridmani * – on the biological market task.

*Pseudochromis*, also known as dottybacks, are a group of small and colorful reef fish from the family Pseudochromidae – the most popular marine ornamental fish. Dottybacks are generalist mesopredators in the wild, meaning they feed mainly on macro-invertebrates and small cryptic species, including other fish (e.g., Ashworth et al. 2014; see Palacios-Narváez et al. 2024). While distinct in many respects, dottybacks and bluestreak cleaner wrasses share some ecological and biological traits. For example, both fish are widespread in the Pacific Ocean – and many are endemic to areas located in the Indian Ocean (e.g., Red Sea) – where they live on coral and rocky reefs and use caves as resting or hiding places, holes, and crevices (Gill 2004; Zander 2017). Both also have an elongated body with bright coloration, show little sexual dimorphism or dichromatism (but see Trigo et al. 2023), and tend to be protogynous hermaphrodites; that is, they first breed as females and some eventually change sex to become males (although male-to-female sex change has also been reported).

Due to their high commercial value, much of the existing research on dottybacks has focused on aquaculture-related biological and behavioral aspects, such as breeding, spawning, larval development, rearing practices, and pair formation (see Chen et al. 2023; Madhu et al. 2016; Mies et al. 2014; Olivotto et al. 2006). Consequently, relatively little is known about the behavioral ecology and cognitive abilities of dottybacks – including our focal species – and comparative studies remain especially rare. Gill (2004) reported no significant dietary differences among dottyback species but did note some behavioral distinctions. Specifically, *P. fridmani* tends to form large groups with closely spaced territories, whereas *P. aldabraensis*, *P. flavivertex*, and other dottybacks are typically highly territorial, maintaining small home ranges either alone, in pairs, or in small groups (“harems”) composed of a male and multiple females, resembling more the social structure of cleaner fish. This behavioral difference is further supported by recent research indicating that *P. fridmani* appears to be more tolerant of conspecifics than other dottyback species (Mies et al. 2014).

In the current study, subjects were tested on three versions of the biological market task (from Prétôt et al. 2016b). All subjects first completed the original plate task, in which the ephemeral and permanent *plates* offering the food rewards differed in both color and pattern. Subjects then received either the color task, where the *food* reward differed in color, or the back task (also known as “cup” task; see Prétôt et al. 2016b), where the food reward was *hidden behind* two plates that differed in color and pattern. In each task, if the subjects reached learning criterion in the initial learning phase, they received a reversal learning test – a measure of cognitive flexibility (Izquierdo et al. 2017). The procedure was identical to the initial learning phase, but the stimuli switched role (i.e., the former ephemeral stimulus became the permanent stimulus, and vice versa). The reversal learning test served two purposes: first, to assess whether dottybacks exhibit cognitive flexibility, a key component of executive functions (Diamond 2013), and second, to evaluate the strength of dottybacks’ preference for the ephemeral stimulus. Specifically, if individuals initially favored the ephemeral stimulus and subsequently shifted their choice to the newly designated ephemeral (formerly permanent) stimulus following reversal, this would suggest a consistent preference and thus an understanding of the utility of selecting the ephemeral stimulus (see Prétôt et al. 2016a, b, 2020; Salwiczek et al. 2012).

We did not have a strong a priori prediction about how dottybacks would perform across the different versions of the biological market task. If the task closely models the specific interaction between cleaner fish and their clients, we would expect all dottybacks to perform poorly in the three versions, regardless of the cue. Conversely, if dottybacks performed significantly better on one task over the other, it could shed light on which cue(s) influence(s) their foraging decisions. Specifically, stronger performance in the color task compared to the plate task would suggest that the color task aligns more closely with the decision-making strategies of opportunistic mesopredators, which often rely on visual cues to locate prey. Following Prétôt et al. (2016b), we had no particular prediction for the effect of concealing the food (back task) on dottybacks’ performance.

## Methods

### Subjects

We tested 14 aquacultured subadult dottybacks from Ocean, Reefs & Aquariums (ORA Farms, sex unknown) at SEA LIFE Aquarium Kansas City. Subjects were five neon dottybacks (*Pseudochromis aldabraensis*), three sunrise dottybacks (*P. flavivertex*), and six orchid dottybacks (*P. fridmani*). Subjects were individually housed and tested in acrylic tanks that were maintained in a closed system with artificial Crystal Sea Marine Mix at 25ppt salinity and using a Reeflo Barracuda Hybrid pump with a system turnover rate of 10 times an hour. The integrated filtration sump contained bio media, 10-micron filter sock mechanical filtration and protein skimmer. Each tank was divided into two compartments via a mesh partition, each containing one subject and supplied with artificial decorations and opaque Schedule 40 polyvinyl chloride (PVC) tubes used as shelters for hiding during the day and sleeping at night. Subjects were tested in the morning and received their daily food intake following a testing session, independent of their performance during the experiment. They were kept under an 8:16 h light/dark cycle using 8 florescent lights and water was maintained at 25 degrees Celsius, pH 8.0-8.4, total ammonia 0ppm, nitrite 0ppm, nitrate 10ppm. We used an all-in-one seafood blend (Frenzy^®^ from LRS Foods) for all the experiments. Subjects were trained to feed off rectangular Plexiglas plates prior to the experiments (for a similar approach, see Prétôt et al. 2016b; Triki et al. 2020).

### General procedure

All subjects were tested in three experiments: the plate task, color task, and back task. At the beginning of each experiment, subjects received a series of familiarization trials, in which they were exposed to the experimental plates holding the food. The familiarization phase ended when the subject ate the food from the plate(s) in four trials total. Following the familiarization phase, subjects were tested in the experimental task. Each task consisted of two phases: an initial learning phase and a reversal learning phase, of up to 200 trials each. If the subject reached learning criterion in the initial learning test (see Learning criterion and analysis section, below), they were then tested in the reversal procedure, where the task contingencies were reversed. All subjects underwent the plate task first (Prétôt et al. 2016b, 2020); half of the subjects were then tested in the color task prior to the back task while the other half were tested in the back task prior to the color task. The plate task design was based on Triki et al. (2020; also see Triki et al. 2018, 2019; Wismer et al. 2014), whereas the color task and the back task were based on Prétôt et al. (2016b).

**Fig. 1:**
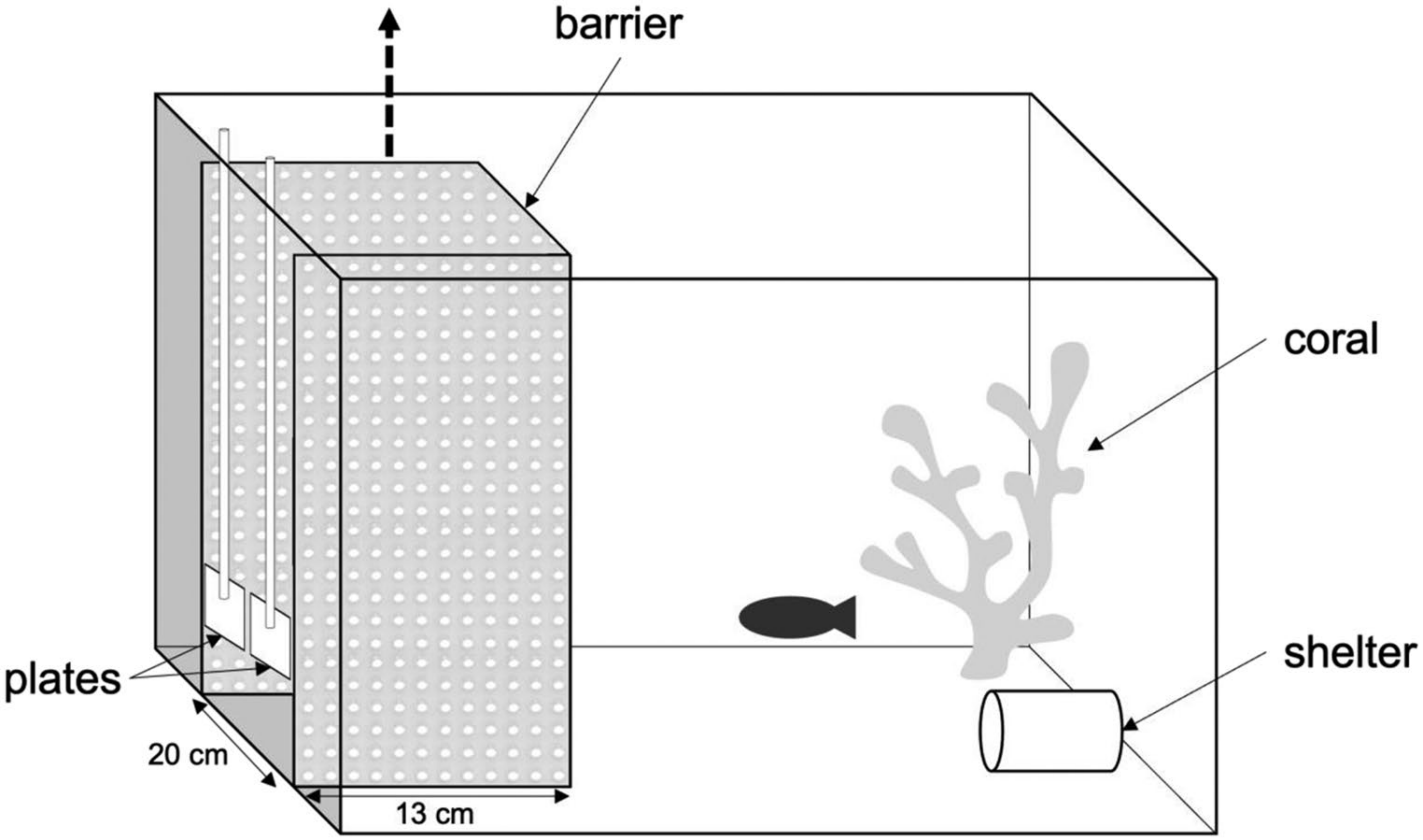
Experimental setup. Before the trial started, the plates carrying the food rewards were placed behind a foldable U-shaped semi-opaque barrier that prevented the fish to directly access the food. The trial started once the barrier was removed out of the tank and the fish could freely access the plates

Each trial consisted of subjects choosing between two plate options, each carrying a same food reward and presented using a handle that allowed the experimenter to move it independently in and out of the tank (see Fig. 1). If the subject ate the food from the ephemeral (optimal) option first, it could also eat the food from the permanent option, resulting in two rewards (i.e., the food-maximizing solution). If the subject ate the food from the permanent (suboptimal) option first, however, the ephemeral option was no longer available, resulting in one reward only. Before a trial started, the experimenter placed the two plates with food at the opposite end of the compartment, behind a foldable U-shaped semi-opaque barrier (20 × 13 cm; Fig. 1) that prevented immediate access to the food.

A trial started when the experimenter removed the barrier out of the tank, allowing the fish to freely access the plates. The side on which each plate was presented for each trial was pseudo-randomized such that each plate was presented an equal number of times on each side with no more than three trials in a row on the same side. If the fish did not eat from either plate within 60 s, the trial ended and was scored as a suboptimal choice. It was not repeated. Those represented approximately 9% of the trials across phases and experiments (initial learning phase: 343/5730 = 6%; reversal learning phase: 414/2240 = 18%). If the fish ate the food from the ephemeral stimulus first within the allotted time, it was given an additional 60 s to eat the food from the permanent plate (for a total of 120 s). Although we reported data for each outcome, we only examined – for the purpose of our analysis – whether subjects made an optimal choice (i.e., choosing the ephemeral stimulus first) or a suboptimal choice (i.e., choosing the permanent stimulus first *or* failing to choose either stimulus within 60 s).

Subjects received one session of 10 trials per day, two to four days a week, for a total of up to 200 trials (unless otherwise noted) *or* until they reached learning criterion (see Learning criterion and analysis section, below). The intertrial interval (ITI) was the time it took subjects to complete one round of trials (i.e., 20–30 min). If the subject reached the learning criterion in the initial learning phase, it was then tested on a reversal learning test, in which the role of each stimulus was switched (i.e., the ephemeral stimulus became the permanent stimulus, and vice-versa; for a similar approach, see Prétôt et al. 2016a, b; Salwiczek et al. 2012). Subjects were tested using a within-subjects design, in which they received the plate task before the other tasks. The order in which the color and back tasks were presented was randomly determined and counterbalanced across subjects within species.

### Plate task

**Fig. 2:**
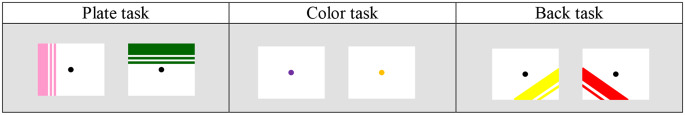
Pairs of stimuli used in the plate, color, and back tasks. Note that for the plate and color tasks, the food was presented on the front of the plate, whereas for the back task, the food was located on the back of the plate

All 14 subjects received the original plate task. In the task, subjects were given a choice between two rectangular plates of similar size (6 × 5 cm) that differed in color and pattern, each carrying a food reward on the front side. One of the plates had three vertical pink stripes, whereas the other had three horizontal green stripes (see Fig. 2). For seven of the subjects, the plate with vertical pink stripes was the ephemeral stimulus and the plate with horizontal green stripes was the permanent stimulus, whereas for the other seven subjects, the plate with horizontal green stripes was the ephemeral stimulus and the plate with vertical pink stripes was the permanent stimulus. All 14 subjects completed the experiment.

### Color task

Twelve out of the 14 subjects tested in the plate task received the color task. Among the two subjects that did not participate, one died before the study started and the other was not tested due to a lack of engagement. In the task, subjects were given a choice between two differently colored food items, each presented on the front of two rectangular plates of similar size (6 × 5 cm), color and pattern. One of the foods was orange and the other was purple (we chose the same color combination as in Prétôt et al. 2016b; Fig. 2). For seven subjects, the orange food was the ephemeral stimulus and the purple food was the permanent stimulus, whereas for the other five subjects, the purple food was the ephemeral stimulus and the orange food was the permanent stimulus. All but three subjects (*n* = 9) completed the experiment. Among the three subjects, two did not finish due to time constraints and one died in the middle of the study (this fish did not participate in the back task).

### Back task

Eleven out of the 14 subjects tested in the plate task received the back task. The three subjects that did not participate were the same as in the color task. In the back task, subjects were given a choice between two rectangular plates of similar size (6 × 5 cm) that differed in color and pattern, each carrying a food reward on the back side. One of the plates had two left diagonal red stripes, whereas the other one had two right diagonal yellow stripes (we chose the same color/pattern combinations as in Prétôt et al. 2016b; Fig. 2). For six of the subjects, the plate with left diagonal red stripes was the ephemeral stimulus and the plate with right diagonal yellow stripes was the permanent stimulus, whereas for the other five subjects, the plate with right diagonal yellow stripes was the ephemeral stimulus and the plate with left diagonal red stripes was the permanent stimulus. All 11 subjects completed the experiment.

### Methodological considerations

While we strove to keep the procedure as similar as possible to the one previously used with cleaner fish (Prétôt et al. 2016b; Triki et al. 2020), we chose to modify the original procedure to facilitate learning in the task and address discrepancies across testing sites. First, as indicated above, the subjects received a series of familiarization trials prior to testing and were allowed extended time to complete each trial. These adjustments were designed to help subjects acclimate to the experimental setup. Second, due to logistic constraints related to testing in a public aquarium, we were unable to separate each tank into an experimental compartment and a resting compartment using a door system (as in Prétôt et al. 2016b). Specifically, the tanks were attached to a rack and the space above those located at the bottom would have been too small to insert and remove doors. Therefore, we opted for a foldable U-shaped semi-opaque barrier that allowed moving it in and out with ease while blocking subjects’ access to the plates prior to the beginning of a trial (Fig. 1).

### Learning criterion and analysis

We used the same learning criterion as in earlier work (Triki et al. 2020). Subjects were considered to have *solved* the task when they reached a statistically significant preference for the ephemeral stimulus within 200 trials, which could be achieved by choosing the ephemeral stimulus in (i) nine or 10 trials out of a session of 10 trials, (ii) eight trials on two consecutive sessions of 10 trials each, or (iii) seven trials on three consecutive sessions of 10 trials each. In contrast, subjects *failed* in the task if they did not develop a preference for the ephemeral stimulus within 200 trials. The learning criterion was the same for the reversal test.

We conducted two analyses. First, we used a two-tailed exact Fisher’s test with mid-p correction to compare the number of individuals who met criterion for preferring the ephemeral to those who did not in the initial learning phase of the tasks (for a similar approach, see Prétôt et al. 2016b). For this analysis, we only included subjects who completed at least two tasks. We also used a two-tailed exact Fisher’s test with mid-p correction to compare the number of individuals who met criterion for preferring the ephemeral stimulus on both initial and reversal learning phases of the tasks between dottybacks and two populations of bluestreak cleaner wrasses previously tested in the tasks – one from the Philippines that received the plate task (Salwiczek et al. 2012) and the other from Moorea that received all three tasks (Prétôt et al. 2016b). Note that our study differed from the cleaner fish studies in two ways; first, all cleaner fish received up to 100 trials, whereas dottybacks were tested in up to 200 trials, and second, Moorean cleaner fish were also considered failing in the tasks if they developed a preference for the *permanent* stimulus (unlike in the other studies). For these reasons, we compared subjects who preferred the ephemeral stimulus to those who failed to do so within 100 trials (whether they preferred the permanent stimulus or showed no preference).

Second, we used logistic mixed-effects models (generalized linear mixed models, GLMMs; Bolker et al. 2009), with the proportion as the response weighed by the number of trials (see Agresti 2003), to compare subjects’ performance across species, tasks, ephemeral stimuli, learning phases, and sessions. For our entry points, we used the proportion (in percent) of ephemeral choices per session (i.e., out of 10 trials). Mixed models were run using the package “lme4” (Bates et al. 2014). We compared our models to models excluding predictors of interest using likelihood ratio tests (LRTs). For the comparison, subject identity was fit as a random effect (intercepts) to control for repeated measures. When the test result was significant, we conducted post-hoc analyses with multivariate *t* (mvt) adjustments using the package “emmeans”. The statistical analyses of our models were conducted with R statistical software (version 4.5.0, 2025-04-11, How About a Twenty-Six; R Core Team 2025).

Our procedure was as follows. We first created a full model, which included our predictor variables of interest: species, task, learning phase, and session. We compared our full model to a null model, which included only our random effect terms (i.e., subject identity), and found that the full model was better than a null model (LRT, χ^2^_6_ = 170.61, *p* < 0.01; see Table S1 in Supplementary Information). Second, we created a model that included our variables of interest and the interaction between task and session (Task x Session) to further assess differences in learning performance across tasks. Finally, to examine whether features of the ephemeral stimulus (e.g., color, pattern) influenced choice behavior during the initial learning phase, we created reduced models for each task that included species, ephemeral stimulus, and their interaction (Species x Ephemeral).

## Results

**Fig. 3:**
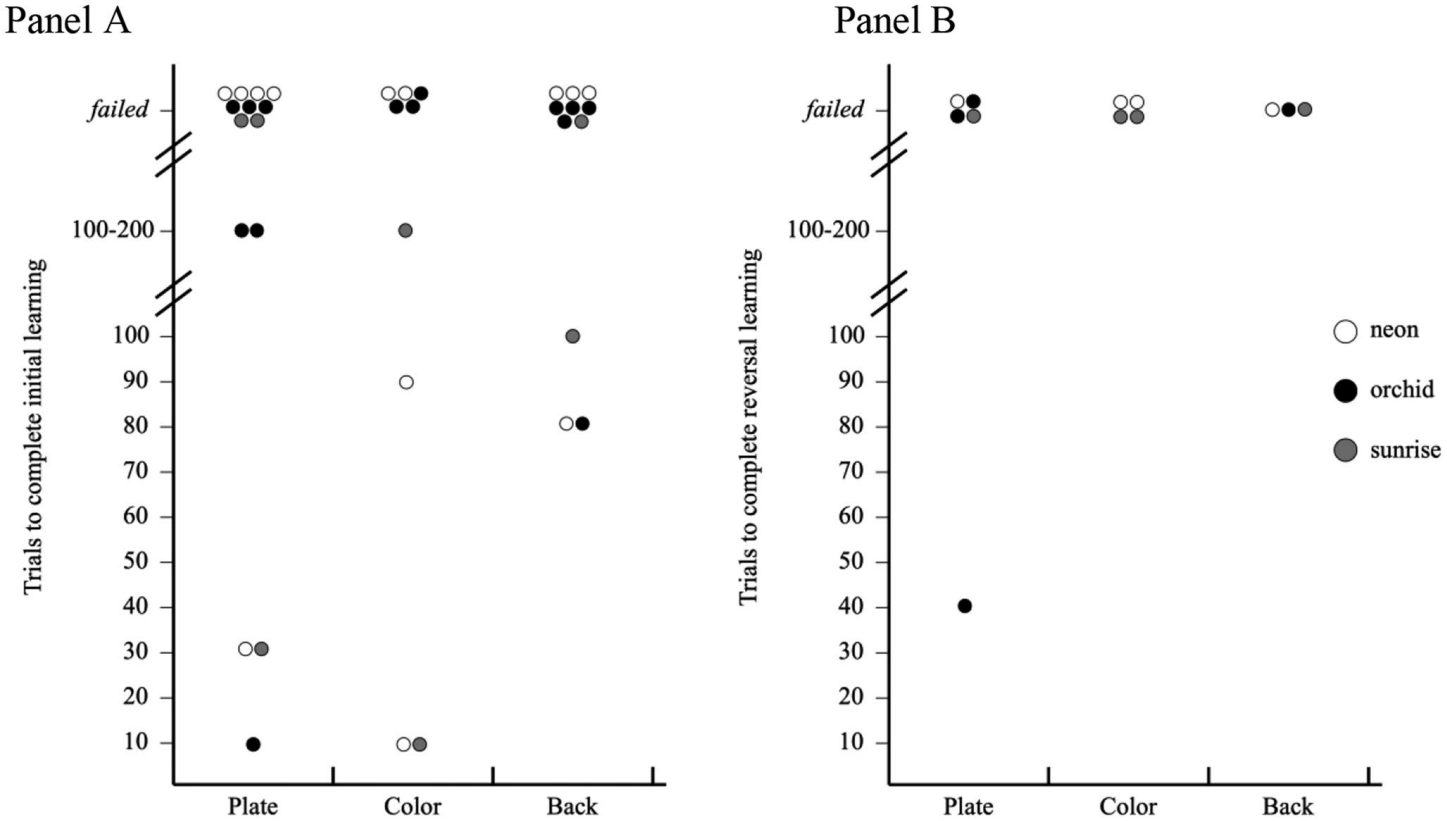
Number of trials needed for subjects to show a preference for the ephemeral stimulus in the initial learning phase (Panel **A**) and reversal learning phase (Panel **B**) of the plate task, the color task, and the back task. Each dot represents one individual; the white dots are neon dottybacks (*Pseudochromis aldabraensis*), black dots are orchid dottybacks (*P. fridmani*), and gray dots are sunrise dottybacks (*P. flavivertex*). The subjects that did not reach the preference criterion within 200 trials are noted “failed”. Note that only data from subjects who completed the tasks are shown in the figure

The number of trials needed for each subject to reach learning criterion across tasks and learning phases are shown in Table 1; Fig. 3. The detailed models are shown in Table S1.

### Plate task

#### Initial learning phase

Three out of 14 subjects (21%) reached learning criterion within 100 trials, and two others (14%) did so within 200 trials. The remaining nine subjects (64%) failed to reach criterion within 200 trials.

#### Reversal learning phase

One out of five subjects (20%) who reached learning criterion in the initial phase did so within 100 trials in the reversal phase (40 trials), whereas the other four subjects (80%) failed to do so within 200 trials.

### Color task

#### Initial learning phase

Three out of nine subjects (33%) who completed the task reached learning criterion within 100 trials, and one (11%) did so within 200 trials. The other five subjects (55%) failed to reach criterion within 200 trials.

#### Reversal learning phase

All four subjects (100%) who reached learning criterion in the initial phase failed to do so within 200 trials in the reversal phase.

### Back task

#### Initial learning phase

Three out of 11 subjects (27%) who completed the task reached learning criterion within 100 trials. The other eight subjects (72%) failed to reach criterion within 200 trials.

#### Reversal learning phase

All three subjects (100%) who reached learning criterion in the initial phase failed to do so within 200 trials in the reversal phase.

**Table 1:** Number of trials needed for each subject to reach learning criterion in the initial learning phase and the reversal phase (when applicable) of the plate, color, and back tasks

Subject		Plate		Color		Back	
ID	species	initial	reversal	initial	reversal	initial	reversal
1	neon	> 200	-	-	-	-	-
2	neon	30	> 200	> 200	-	> 200	-
3	orchid	> 200	-	> 200	-	> 200	-
4	orchid	10	40	> 200	-	> 200	-
5	orchid	> 200	-	> 50*	-	-	-
6	sunrise	30	> 200	-	-	-	-
7	neon	> 200	-	10	> 200	80	> 200
8	neon	> 200	-	> 200	-	> 200	-
9	neon	> 200	-	90	> 200	> 200	-
10	orchid	> 200	-	> 200	-	> 200	-
11	sunrise	> 200	-	140	> 200	> 200	-
12	sunrise	> 200	-	10	> 200	100	> 200
13	orchid	160	> 200	> 180*	-	> 200	-
14	orchid	170	> 200	> 190*	-	80	> 200

*Note*: Subjects who did not reach learning criterion in the initial learning phase were not tested on the reversal phase. Subjects who were not tested in the task are marked with a hyphen (-) and those who did not complete the task are marked with an asterisk (*) following the number of trials received before the task ended. See General procedure section for explanations on task incompletion

### Plate task vs. color task

Nine subjects completed both plate and color tasks. We found no difference in the number of subjects who reached learning criterion in the initial learning phase between tasks, with two subjects reaching learning criterion within 200 trials in the plate task and four subjects reaching learning criterion within 200 trials in the color task (Fisher’s test: *p* = 0.38).

### Plate task vs. back task

Eleven subjects completed both plate and back tasks. We found no difference in the number of subjects who reached learning criterion in the initial learning phase between tasks, with four subjects reaching learning criterion within 200 trials in the plate task and three subjects reaching learning criterion within 200 trials in the back task (Fisher’s test: *p* = 0.68).

### Color task vs. back task

Nine subjects completed both plate and back tasks. We found no difference in the number of subjects who reached learning criterion in the initial learning phase between tasks, with four subjects reaching learning criterion within 200 trials in the color task and two subjects reached learning criterion within 200 trials in the back task (Fisher’s test: *p* = 0.38).

### Comparisons across tasks, phases, and species

Our first model revealed that subjects’ proportion of ephemeral choices was predicted by task (LRT, χ^2^_2_ = 29.09, *p* < 0.01; Table S1) and phase (LRT, χ^2^_1_ = 123.72, *p* < 0.01), but not species (LRT, χ^2^_2_ = 0.61, *p* = 0.74) or session (LRT, χ^2^_1_ = 0.74, *p* = 0.39). Post-hoc comparisons showed that subjects chose the ephemeral stimulus more often in the back task than in the plate task (estimate = 0.25, se = 0.06, *p* < 0.01) and the color task (estimate = 0.30, se = 0.06, *p* < 0.01). However, subjects did not show any difference between color and plate tasks (estimate = -0.06, se = 0.06, *p* = 0.63). Subjects chose the ephemeral stimulus more often in the initial learning phase than in the reversal learning phase (estimate = 0.68, se = 0.06, *p* < 0.01).

Our second model confirmed an additional interaction between task and session (LRT, χ^2^_2_ = 9.65, *p* < 0.01; Table S1). Post-hoc comparisons indicated that subjects chose the ephemeral stimulus more often across sessions in the back task than in the plate task (estimate = 0.25, se = 0.06, *p* < 0.01) or the color task (estimate = 0.30, se = 0.06 *p* < 0.01). However, subjects did not show any difference between color and plate tasks (estimate = -0.05, se = 0.06, *p* = 0.66). 

Finally, the reduced models revealed an interaction between species and ephemeral stimulus in the color task (LRT, χ^2^_2_ = 6.26, *p* = 0.04). Although post-hoc comparisons did not yield statistically significant differences, a marginal trend was observed, with sunrise dottybacks choosing the orange ephemeral food more frequently than orchid dottybacks (estimate = 2.41, se = 1.11, *p* = 0.07; all other *p*s ≥ 0.13). No main effects or interactions were found in the other tasks (plate task: LRT, χ_2_ = 2.91, *p* = 0.23; back task: LRT, χ^2^_2_ = 1.30, *p* = 0.52).

### Dottybacks vs. cleaner wrasses from previous studies

For the plate task, fewer dottybacks met criterion for preferring the ephemeral stimulus in the initial phase (3 out of 14 subjects) than did Philippine cleaner fish (6 out 6 subjects; Fisher’s test: *p* < 0.01) and Moorean cleaner fish (9 out of 14 subjects; Fisher’s test: *p* = 0.03). In the reversal phase, fewer dottybacks preferred the ephemeral stimulus (1 out of 5 subjects) than did Philippine cleaner fish (6 out of 6 subjects; Fisher’s test: *p* = 0.02), but dottybacks did not differ from Moorean cleaner fish (2 out of 9 subjects; Fisher’s test: *p* = 0.96).

For the color task, marginally fewer dottybacks preferred the ephemeral stimulus in the initial phase (3 out of 11 subjects) than did Moorean cleaner fish (6 out of 8 subjects; Fisher’s test: *p* = 0.06). In the reversal phase, however, dottybacks (0 out of 4 subjects) did not differ from Moorean cleaner fish (0 out of 6 subjects; Fisher’s test: *p* = 1.00).

For the back task, dottybacks did not differ from Moorean cleaner fish in either the initial phase (3 out 11 dottybacks vs. 3 out of 8 Moorean cleaner fish; Fisher’s test: *p* = 0.67) or the reversal phase (0 out of 3 subjects each; Fisher’s test: *p* = 1.00).

## Discussion

In the biological market task, subjects are given a choice between two food options presented simultaneously: if they eat from the ephemeral stimulus first, they can also eat from the permanent stimulus, whereas eating from the permanent stimulus first leads to the loss of the ephemeral reward. Previous work showed that bluestreak cleaner wrasses outperformed several non-human primate species on the task, presumably because the experiment simulates a natural foraging dilemma encountered by cleaner fish but not primates. It has been argued that one possible explanation for the high performance of cleaner fish in the task is that their ability to solve it is linked to very specific ecological conditions that are not met in any other species. Although several vertebrates have been tested on the task over the past decade, empirical evidence from other fish species is lacking (but see Ranucci et al. 2024). Here, we provided initial evidence from *non-*cleaner fish on the biological market task. Our study revealed three main findings.

First, dottybacks performed poorly across all versions of the task. These results add to the extended list of species underperforming in the biological market task and further indicate that cleaner wrasses’ skill at the task is likely linked to specific ecological conditions not met in other species. Dottybacks are natural mesopredators who feed on small invertebrates (e.g., crustaceans, mollusks, annelids), fish, and fish larvae (although differences in the prey composition and size of trophic niche exist between species; Palacios-Narváez et al. 2024). Importantly, preys differ in their level of mobility, with some forming dense immobile aggregations in the bottom of the water (e.g., benthic copepods), whereas others must be hunted in open waters (e.g., pelagic copepods, fish), which may roughly correlate with cleaner fish’ stationary permanent clients and mobile ephemeral clients, respectively. However, unlike cleaner fish who actively and selectively search for parasites on client bodies, dottybacks are opportunistic feeders who likely rely on less sophisticated foraging decision rules in the presence of both permanent and ephemeral preys (i.e., focusing on whatever prey is more readily available). Consequently, dottybacks may have performed poorly in the task because they differ from cleaner fish in the way they approach and engage with food sources. Supporting this view, we found that dottybacks were less likely to solve the original plate task than cleaner fish, and the difference was less pronounced or inexistent in the other tasks, suggesting that the plate task more closely simulates the specific ecological conditions faced by cleaner fish in their natural habitat.

It is also worth noting that most subjects who eventually solved the plate task failed to solve the subsequent tasks (3 out of 4 subjects). Although we draw this observation from a limited sample and cannot rule out possible carry-over effects, this is an interesting finding because it indicates that previous experience with the task might not be a key factor of success. These results further indicate that subjects might perceive the versions of the task differently and treat them independently. Previous work with primates indicates that variation in task performance could be due to differences in species’ cognitive testing history (Prétôt et al. 2016b). Although this could not apply to our dottybacks – who had no prior testing experience – it is possible that our finding reflects some aspects of the subjects’ life history, including the complexity of their environment. Supporting this, previous studies found that cleaner fish from areas with high population densities were more successful in solving the biological market task than those from areas with low population densities (Triki et al. 2018, 2019). In addition, there is evidence that environmental enrichment or change enhances cognitive performance in various tasks (e.g., Arechavala-Lopez et al. 2020; Kotrschal and Taborsky 2010; Lucon-Xiccato et al. 2023; Montalbano et al. 2022; Salvanes et al. 2013; Shen et al. 2023; for a review, see Ebbesson and Braithwaite 2012). In the case of our dottybacks, the fact they originated from aquaculture may have inhibited their problem-solving skills due to easier access to food and the absence of consequences following poor foraging decisions. Together, these results indicate that performance in the task is likely driven by a unique combination of environmental and experiential influences on species’ decision-making behavior.

Second, our models revealed subtle differences in cognitive performance between tasks. Importantly, dottybacks chose the ephemeral stimulus more often in the back task than in the other tasks. These results partly align with those of capuchin monkeys tested previously (Prétôt et al. 2016b), indicating that the difficulty of non-cleaner species to solve the task might be due to the prepotent response associated with the presence of visible food rewards. In addition, they further suggest that concealing the food rewards caused subjects to act less impulsively, thus facilitating optimal foraging in the task. We caution, however, against strong interpretations of these results, because of the limited sample size and the low number of subjects who succeeded in the task.

Another important observation from the task comparisons is that dottybacks’ performance did not improve in the color task as compared to the plate task. On one hand, this was a surprising result, given that the color task was originally designed to resemble more foraging decisions faced by opportunistic feeders like dottybacks, who focus on the food itself (e.g., the food color) to distinguish between permanent and ephemeral food sources, than those faced by selective feeders like cleaner fish, who rather focus on the substrate carrying the food (e.g., the plate color/pattern) to distinguish among food sources. On the other hand, however, if the difficulty to solve the task arises primarily from its underlying mechanism (i.e., the contingencies) rather than the nature of the cues involved, then dottybacks would be expected to perform poorly across all cue conditions – a pattern in line with our finding.

Third, dottybacks performed significantly worse in the reversal learning phase than in the initial learning phase. These results are interesting, because they emphasize the difficulty of subjects to learn the new contingencies after the reversal. They are also consistent with previous findings showing that fish perform generally worse in reversal tests than in initial learning tests (e.g., Warren 1960; Lucon-Xiccato and Bisazza 2014). Interestingly, dottybacks performed as poorly as Moorean cleaner fish (Prétôt et al. 2016b) but significantly lower than the Philippine population (Salwiczek et al. 2012). Together, these findings highlight population- and species-level variation in cognitive flexibility among coral reef fish, and emphasize the importance of examining further the role of cognitive flexibility on species’ decision-making behavior.

Finally, it is possible that some aspects of our procedure may have unintentionally influenced subjects’ performance, potentially accounting for some of the observed results. Two factors in particular – habituation and motivation – may help explain dottybacks’ poor performance. With regard to habituation, although the procedure was designed to enhance engagement with the task, subjects failed to respond in 9% of the trials – a non-trivial proportion. This was further supported by the observation that dottybacks were generally slow to initiate trials (L. Prétôt, personal observation). In terms of motivation, it is possible that the incentive structure of the task was insufficient. Specifically, the inclusion of familiarization trials may have resulted in subjects receiving enough food prior to testing, thereby reducing motivation to perform during experimental trials. Clearly, future research is needed to better understand dottybacks’ natural tendencies and acclimation requirements in research settings.

An additional limitation of our experimental design is that successful performance was highly dependent on visual discrimination. As a result, the current procedure does not account for the possibility that subjects may rely on other sensory modalities, such as olfaction or chemical cues, to guide their decisions. While dottybacks are generally described as visual passive predators, studies suggest that certain species that actively hunt – such as the brown dottyback (*Pseudochromis fuscus*) – make extensive use of olfactory cues during foraging (Cripps et al. 2011; Natt et al. 2017). Given the small spatial scale of dottybacks’ hunting behavior and the structural complexity of coral reef habitats, which can limit visual access, olfactory cues might play an important role in prey detection for our focal species as well. Despite this potential sensory mismatch, several subjects reached the learning criterion, and at least one individual succeeded in both the initial and reversal learning phases. This suggests that dottybacks possess, at a minimum, the cognitive abilities necessary to solve the task under the current design. Nevertheless, we encourage future studies to explore modified versions of the biological market task that better reflect dottybacks’ natural foraging strategies. For example, the ephemeral stimulus could be made to “move” at the start of each trial, simulating an escaping prey item. This would create a more ecologically valid context in which dottybacks are asked to choose between a stationary prey (e.g., benthic copepods) and a mobile, potentially fleeing prey (e.g., small fish), akin to the approach used by Prétôt et al. (2016a).

In summary, dottybacks’ poor performance in the biological market task mirrors that of other non-cleaner species tested to date, reinforcing the idea that the task is intrinsically difficult – likely due to its basis in the specialized interactions between cleaner wrasses and their clients. This study offers initial evidence that non-cleaner fish also struggle with the task across multiple versions. Our findings contribute to the growing literature on the biological market task and highlight the need to test a broader range of species that both resemble and differ from cleaner fish in their ecological and biological characteristics. At the same time, refining experimental methods to accommodate diverse sensory and cognitive systems will be crucial for unraveling the factors that drive success in this intriguing, ecologically rooted paradigm.

## Electronic supplementary material

Below is the link to the electronic supplementary material.

Supplementary Material 1

## Data Availability

The data and R code are available at the following link: https://osf.io/vmz5a/?view_only=a983aa773b4242d1811ef6c8baf14dae.
