# Supplementary material for: From learning to reversal learning: How non-cleaner fish tackle the biological market task
Source: Anim Cogn. 2025 Jul 24;28(1):61. doi: 10.1007/s10071-025-01983-w (PMC12289785; doi:10.1007/s10071-025-01983-w)
Supplement: Supplementary file 1 — Supplementary Material 1 [file 10071_2025_1983_MOESM1_ESM.docx]

**Supplementary Information (SI)**

Title: *From learning to reversal learning: How non-cleaner fish tackle the biological market task*

Journal: *Animal Cognition*

Authors: *Laurent Prétôt, Hannah Miller, and Kayla Leyden*

Corresponding author: *L. Prétôt, Department of Psychology and Counseling, Pittsburg State University, Pittsburg, KS 66762, U.S.A. ORCID ID: https://orcid.org/0000-0002-9936-5506*

**Table S1.** Estimate and standard error (s.e.) of fixed effects in mixed models predicting subjects’ preference for the ephemeral option relative to the permanent option (in percent).

|  | **Null** | **Full without interaction** | **Full with interaction** | **Reduced plate task** | **Reduced color task** | **Reduced back task** |
| --- | --- | --- | --- | --- | --- | --- |
| (Intercept) | -0.51 (0.10)^***^ | -0.26 (0.16) | -0.24 (0.17) | -0.36 (0.38) | 0.14 (0.24) | -0.08 (0.12) |
| Species: orchid |  | -0.16 (0.21) | -0.16 (0.21) | -0.63 (0.54) | -0.33 (0.30) | -0.15 (0.15) |
| Species: sunrise |  | -0.12 (0.26) | -0.12 (0.26) | 0.50 (0.75) | 2.08 (1.12) | -0.04 (0.20) |
| Task: back |  | 0.25 (0.06)^***^ | 0.37 (0.11)^**^ |  |  |  |
| Task: color |  | -0.06 (0.06) | -0.25 (0.12)^*^ |  |  |  |
| Phase: reversal |  | -0.68 (0.06)^***^ | -0.68 (0.06)^***^ |  |  |  |
| Session |  | -0.00 (0.00) | -0.01 (0.01) |  |  |  |
| Back x Session |  |  | -0.01 (0.01) |  |  |  |
| Color x Session |  |  | 0.02 (0.01)^*^ |  |  |  |
| Ephemeral plate: pink striped |  |  |  | 0.48 (0.62) |  |  |
| Orchid x pink striped |  |  |  | 1.06 (0.84) |  |  |
| Sunrise x pink striped |  |  |  | -0.55 (1.02) |  |  |
| Ephemeral color: purple |  |  |  |  | -0.46 (0.39) |  |
| Orchid x purple |  |  |  |  | -0.37 (0.47) |  |
| Sunrise x purple |  |  |  |  | -2.72 (1.21)^*^ |  |
| Ephemeral back: yellow striped |  |  |  |  |  | 0.26 (0.18) |
| Orchid x yellow striped |  |  |  |  |  | -0.01 (0.24) |
| Sunrise x yellow striped |  |  |  |  |  | 0.35 (0.33) |
| AIC | 3564.73 | 3406.12 | 3400.47 | 815.65 | 715.37 | 683.31 |
| BIC | 3574.09 | 3443.57 | 3447.28 | 839.41 | 737.20 | 705.89 |
| Log Likelihood | -1780.37 | -1695.06 | -1690.23 | -400.83 | -350.69 | -334.66 |
| Number of trials | 797 | 797 | 797 | 220 | 167 | 186 |
| Number of subject (ID) | 14 | 14 | 14 | 14 | 12 | 11 |
| Variance: subject ID (Intercept) | 0.12 | 0.11 | 0.11 | 0.40 | 0.08 | 0.01 |

*Note*. Baselines for factors were as follows: species = neon, task = plate, learning phase = initial, ephemeral plate = green striped, ephemeral color = orange, ephemeral back = red striped. The table also shows goodness-of-fit statistics.

^***^p < 0.001; ^**^p < 0.01; ^*^p < 0.05
